# Strategies for increasing accrual in cancer clinical trials: What is the evidence?

**DOI:** 10.1002/cam4.7298

**Published:** 2024-05-21

**Authors:** Margo Michaels, Elisa S. Weiss, Maria Sae‐Hau, Dora Illei, Bethany Lilly, Leah Szumita, Brian Connell, Marialanna Lee, Eric Cooks, Melissa McPheeters

**Affiliations:** ^1^ Health Access and Action Consulting Newton Massachusetts USA; ^2^ The Leukemia & Lymphoma Society New York New York USA; ^3^ RTI International Research Triangle Park North Carolina USA

**Keywords:** accrual, clinical trials

## Abstract

**Introduction:**

Despite the importance of clinical trial participation among cancer patients, few participate—and even fewer patients from ethnic and racial minoritized groups. It is unclear whether suggested approaches to increase accrual are successful. We conducted a scoping review to identify evidence‐based approaches to increase participation in cancer treatment clinical trials that demonstrated clear increases in accrual. Notably, more stringent than other published reviews, only those studies with comparison data to measure a difference in accrual rates were included.

**Methods:**

We searched PubMed/MEDLINE, Embase, CINAHL, and Web of Science for English‐language articles published from January 1, 2012, to August 8, 2022. Studies were included if they were conducted in the United States, described single or multicomponent interventions, and provided data to measure accrual relative to baseline levels or that compared accrual rates with other interventions.

**Results:**

Sixteen articles were included: six with interventions addressing patient barriers, two addressing provider barriers, seven describing institutional change, and one describing policy change. Key themes emerged, such as a focus on patient education, cultural competency, and building the capacity of clinics. Few studies provide comparative accrual data, making it difficult to identify with certainty any effective, evidence‐based approaches for increasing accrual. Some patient‐ and system‐level interventions studies showed modest increases in accrual primarily through pre‐post measurement.

**Conclusion:**

Despite an extensive body of literature about the barriers that impede cancer treatment trial accrual, along with numerous recommendations for how to overcome these barriers, results reveal surprisingly little evidence published in the last 10 years on interventions that increase accrual relative to baseline levels or compared with other interventions. As clinical trials are a primary vehicle through which we improve cancer care, it is critical that evidence‐based approaches are used to inform all efforts to increase accrual. Strategies for increasing participation in cancer clinical trials must be developed and rigorously evaluated so that these strategies can be disseminated, participation in trials can increase and become more equitable, and trial results can become more generalizable.

## 1 INTRODUCTION

Receiving cancer treatment within a clinical trial is considered high‐quality care,^1^, ^2^ but access to and participation in these trials is low and inequitable. Data show that longer survival and lower mortality are correlated with clinical trial participation.^3^ However, less than 8% of people with cancer participate in a cancer treatment clinical trial.^4^ Moreover, it is estimated that only about 15% of those participating are from racial and ethnic minoritized groups^4^, ^5^ even though these groups comprise more than 40% of the US population. This underrepresentation is particularly concerning given the higher incidence of cancers, and known inequities in outcomes, among Black/African American and Hispanic/Latino populations in particular.^6^ For decades, data have consistently shown that groups underrepresented in cancer treatment trials include people from ethnic and racial minoritized groups,^7^, ^8^, ^9^ people with low incomes,^9^, ^10^ those who live in rural areas,^11^ people who are aged 70 years and older,^12^, ^13^, ^14^ and adolescents and young adults aged 15–39 years.^15^

It is critical to improve equity in access to cancer treatment clinical trials and in particular, address barriers faced by racial and ethnic minoritized groups and other underrepresented populations; these barriers occur at the levels of the patient, clinician/research team, institution, and system.^16^, ^17^ For example, often‐cited barriers facing patients generally include awareness, attitudes, and concerns about travel and cost.^5^ Further upstream barriers facing clinicians include failure to prescreen patients for eligibility, biases about discussing trials as an option for treatment^18^ and assumptions about a patient's treatment preferences.^19^ At the health systems level, cancer treating facilities may face limited availability of clinical trials, restrictive eligibility criteria in available trials,^20^ limited staffing and infrastructure capacity and capability,^21^, ^22^, ^23^ ineffective patient screening and enrollment practices,^16^, ^24^, ^25^, ^26^, ^27^, ^28^, ^29^, ^30^, ^31^ or poor community engagement.^5^, ^30^

Across all potential barriers, however, there has been very little documentation of evidence showing what works to increase accrual. We sought to identify any available evidence about approaches or interventions for increasing cancer treatment trial accrual rates, overall or for any particular population group, focusing only on studies that provided evidence that could be used to assess whether an approach improved accrual, and if so, by how much. Additionally, we sought to determine whether there was evidence to indicate that the effectiveness of interventions to increase accrual in cancer treatment clinical trials differed by any subgroup, such as by race/ethnicity, geographical location, or cancer type.

This scoping review differs from other recent reviews of approaches to increase US cancer treatment trial accrual in important ways. Other reviews, concluding that certain approaches have improved accrual, utilized different definitions of accrual^32^/participation, or different inclusion criteria (e.g., including treatment, screening and cancer control studies).^33^, ^34^ Different from many other reviews, this scoping review requires that studies present comparison data to measure a difference between intervention groups or change over time from baseline in accrual rates in the same population. We believe these data are critical to establish the effectiveness of any intervention.

## 2 METHODS

We conducted a scoping literature review by searching PubMed/MEDLINE, Embase, CINAHL, and Web of Science for English‐language articles published from January 1, 2012, to August 8, 2022, from the United States. The complete search strategy for all data sources is provided in Appendix. The search strategy included a number of cancer‐related terms and required that studies focused on accrual of participants into cancer treatment trials.

Three researchers completed title and abstract review for inclusion, with the inclusion or exclusion determined by consensus when there was initial disagreement. Two researchers then completed the full article review for inclusion, where disagreements were resolved via consensus.

Studies were included if they were conducted in the United States, described single or multicomponent interventions, and provided data to measure accrual relative to baseline levels and/or data that compared accrual rates with other interventions. The outcome of focus was changes in rate of accrual, defined as the number or proportion of cancer patients that enroll in a trial over a specific time frame. Studies that focused primarily on pediatric patients were excluded; trials that focused on adolescents, young adults or adults were included.

One researcher abstracted all of the full articles included. Information about study population, sample size, intervention type, outcomes of interest, methods, and study results was abstracted from all full articles included. Guided by existing literature,^4^, ^35^, ^36^ articles were also categorized into four study types: articles describing institutional change, studies addressing patient barriers, articles addressing provider barriers, and articles describing policy changes at the state level.

Only four studies were randomized controlled trials (RCTs); all other studies were pre‐post analyses with significant design weaknesses.

## 3 RESULTS

### 3.1 Article characteristics

**FIGURE 1:**
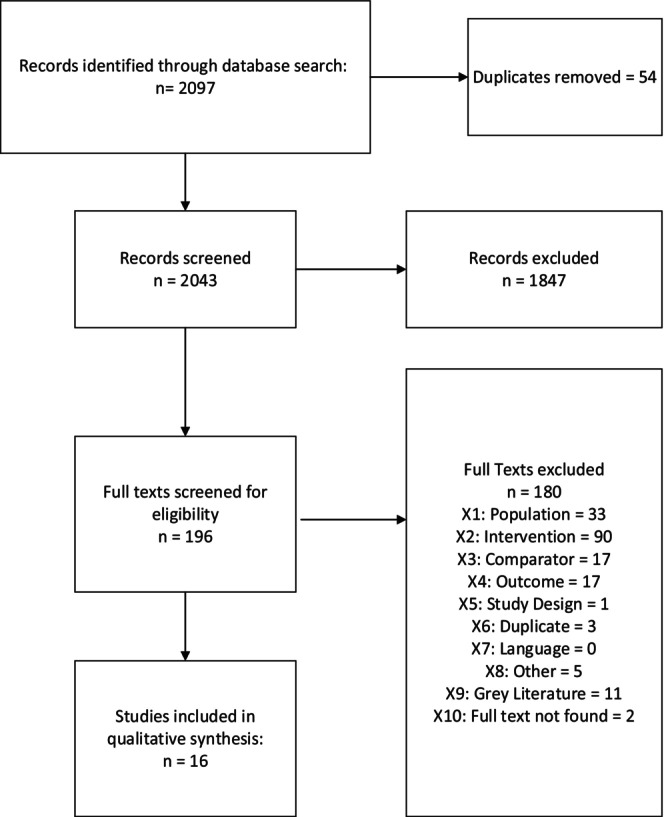
Identification of articles.

We identified 2043 nonduplicate records through our initial search. Most (*n* = 1847) were excluded at the title and abstract phase. Of the 196 articles screened at the full‐text phase, the most common reason for exclusion was that the paper did not describe or evaluate a specific intervention. The second most common reason for exclusion was that the study was conducted in a population that did not meet our inclusion criteria (e.g., not in the United States). Furthermore, many studies did not provide any comparative data (including pre‐post) that would allow us to quantify a change in accrual. Figure 1 provides a summary of all articles included in the scoping review.

We identified 16 papers that met our inclusion and exclusion criteria. We classified them as six studies addressing patient barriers, one article addressing provider barriers, eight articles describing systems‐level change (primarily at the clinic or hospital level), one article focused on clinicians and research staff, and one article describing policy changes at the state level. Several key themes emerged among these interventions, such as a focus on patient education, a focus on cultural competency, and a focus on building the capacity of clinics to improve trial accrual.

Most studies (12 of 16) did not specify cancer types. Still, two focused on breast cancer, one on prostate cancer, one on gynecologic cancers generally, and one on cancer in adolescents and young adults (no specific type).

## 4 INTERVENTIONS TARGETING SYSTEMS‐LEVEL CHANGES

Eight articles described institutional changes made to improve clinical trial enrollment (Table 1). These changes included active prescreening and trial matching, multicomponent programming, collaborative clinical programs, and molecular tumor boards.

**TABLE 1:** Studies of systems‐level changes.

Study/Type/*N*	Patient population/Setting	Comparison/intervention	Results
Rimel et al.^37^ Pre‐post *N* = 507	Gynecologic oncology patients at an academic medical center	Web‐based registry with a trial matching mechanism and online consent versus paper‐based registry	No patients from either cohort were eligible for a therapeutic trial, so it was not possible to compare effectiveness No effect on overall percentage that enrolled in studies (15% of eligible patients; *p* = 0.934)
Wu et al.^65^ Pre‐post QI implementation Total N unspecified	Oncology patients at a safety net hospital affiliated with an academic health center	QI initiative implemented in an academic clinical trials office. A full‐time employee identified patients potentially eligible for a clinical trial through a combination of the EHR, tumor board, and cancer registry and routed them to physicians for trial teaching and enrollment	Prescreening was associated with a more than 4× increase in enrollment In the 2 years prior, 31 patients were enrolled in clinical trials In the 24 months after implementation, 255 patients were identified, and 143 (56.1%) enrolled
Farhangfar et al.^39^ Pre‐post Sample size not provided	Patients at a multisite regional cancer system	Standardization of molecular profiling and knowledge management system to inform clinicians. Analysis compared use of molecular profiling (MP) and molecular tumor board (MTB) before and after system implementation and compared accrual among those with MP versus those with MP + MTB	More patients with MTB + MP (28%) enrolled in clinical trials compared with 15% for those with MP only. However, the decision to engage with MTB was driven by the clinician, so there is no indication that the two groups are comparable
Mobley et al.^40^ Retrospective Analysis *N* = 11,794	Patients at a comprehensive cancer center	Reviewed records for all patients seen over a 5‐year period and compared the rate of enrollment in clinical trials by whether their case had been discussed at a tumor board	4.1% of patients discussed at a tumor board were consented to a trial compared with 2.8% of those not discussed (*p* < 0.01) Likelihood of discussion at a tumor board did not vary by insurance or rural/urban status No data on other potential differences between groups (e.g., cancer stage)
Trant et al.^41^ Pre‐post *N* = not specified (community level)	Breast cancer patients at an academic health center serving an underserved, urban population	Multicomponent approach, including community outreach by physicians and community health workers, executive council representation, grand rounds, and didactic lectures with healthcare providers at FQHCs	Significant increase in the proportion of African American and Hispanic patients participating in trials from 95/750 (12.7%) in 2016 to 155/944 (16.4%) in 2018 (*p* = 0.03)
Anwuri et al.^35^ Pre‐post Sample size not provided	Patients at a comprehensive cancer center	Multicomponent structural intervention to create centralized organizational accountability for clinical trial recruitment of minority patients, defined as African American and Other. The intervention focused on investigators, including leadership support, policy change, process control, data analysis and reporting, and follow‐up with clinical investigators	Although the number of minority patients participating in clinical trials increased over the 5‐year study period, the proportion of minority patients did not
Madsen et al.^42^ Retrospective pre‐post *N* = 1370	Men diagnosed within 6 months with nonmetastatic localized prostate cancer at a comprehensive cancer center	Tailored summaries of recommended clinical trials were provided to patients and reviewed with an APN at each clinic visit	Enrollment in novel studies (investigational treatments and systemic agents) increased from 6% to 15%, and enrollment in procedural trials decreased from 8% to 2%
Shaw et al^43^ Retrospective pre‐post *N* = 49	Newly diagnosed AYA (ages 15–40) cancer patients with multiple cancer types (included both solid and hematologic) who were referred from area hospitals to the joint AYA program from 2006 to 2010	A joint program of pediatric and medical oncology programs in which pediatric and adult clinicians collaborated to evaluate and provide consultation to AYA patients with the primary goal of increasing clinical trial accrual at both sites Multidisciplinary meetings were held quarterly where pediatric and adult teams shared information about available trials	Among AYA patients referred to the program and treated at the adult hospital, trial enrollment increased from 4% to 32%; *p* < 0.001 No increase was seen among those referred to and treated at the children's hospital

Abbreviations: APN, advanced practice nurse; AYA, adolescent and young adult; EHR, electronic health record; FQHC, Federally Qualified Health Center; MP, molecular profiling; MTB, multidisciplinary tumor board; QI, quality improvement.

### 4.1 Active prescreening and trial matching

Two studies examined implementation of screening strategies to better identify potentially eligible patients through prescreening and trial matching practices, with some reported success.

Rimel et al.^37^ compared a web‐based online registry and trial matching program to a traditional paper‐based registry for gynecological cancer studies. Introduction of the web‐based registry was associated with a substantial increase in the number of women participating in the registry, from 5.4 women per month to 23 women per month. Patients who enrolled in the registry through the web‐based system were more likely to be non‐White than those who enrolled through the paper‐based system (25% vs. 15%, respectively; *P* < 0.001). Of the women who enrolled in the web‐based registry, 82% were matched with at least one study, and 15% of those women enrolled but no patients were eligible in the first year for an intervention study. Although the intervention was associated with substantially increased participation in the web‐based registry, the paper did not provide data to establish that increased participation in the registry was associated with increased trial accrual.

A quality improvement initiative at a public hospital conducted manual screening by one full‐time employee to identify eligible cancer patients through tumor boards, cancer registries, and clinic schedules.^38^ In the 24 months prior to the program, 31 patients were enrolled in clinical trials. In the 24 months during the study period, 255 patients were identified as potentially eligible for trial enrollment, of whom 143 were successfully initially enrolled (though 15% of those enrolled patients were ultimately found ineligible after enrollment). No data were provided regarding why patients were determined to be ineligible after trial enrollment. The increase in enrollment was 4.6 times over baseline.

### 4.2 Patient trial matching via molecular tumor boards

We included two studies that examined the use of molecular tumor boards; these studies may provide some indication of the potential for another form of patient matching when made available. Farhangfar et al.^39^ compared clinical trial enrollment of patients who underwent molecular profiling alone with those who completed both molecular profiling and the addition of a molecular tumor board through a clinical genomics program at a multisite cancer center with locations across North and South Carolina. Among a cohort of 191 patients reviewed by the tumor board during the first 2 years of the program, 43% who both underwent molecular profiling and review by the tumor board were consented to a clinical trial, and 28% enrolled, compared with only 15% of patients who received only molecular profiling.

Mobley^40^ examined the impact of multidisciplinary tumor board (MTB) meetings on consent rate for cancer treatment trials. Patients included in the study were new oncology patients at the University of Iowa Health Care oncology clinics. Of the 11,794 patients included in the study period (2011–2015), 2225 (18.9%) were discussed at MTB meetings. Of these, 92 (4.1%) consented to a clinical trial. Patients whose cases were discussed at MTB meetings gave consent to participate in clinical trials at a higher rate than those whose cases were not discussed (4.1% vs. 2.8%, respectively). However, 76 of the total consented patients in both groups (*n* = 357 across groups, with 92 in the MTB group and 265 in the non‐MTB group) did not enroll in a trial for a variety of reasons, including change in diagnosis or disease stage between time of consenting and start of the study, patient preference, and other reasons. This study may be confounded by the fact that physicians chose which patients should be discussed at tumor board meetings and did not provide data on clinical differences between patient groups.

### 4.3 Multicomponent programming

Three multicomponent studies used pre‐post designs with mixed results. In a multicomponent, community‐oriented program^41^ at an academic cancer center in an urban, low‐income setting, a team implemented community outreach through physicians and health workers, ensured representation by researchers on the cancer center's executive council, provided grand rounds to raise awareness among investigators, and provided didactic lectures for community providers. There was a significant increase in the proportion of cancer patients enrolled in clinical trials who were Black or Hispanic between 2016 and 2018 (12.7% vs. 16.4%; *p* = 0.0325), despite no significant differences in the rate of clinical trial invitation by race/ethnicity.

Anwuri et al.^35^ reported on an organizational change model to increase accountability among researchers to improve the accrual of patients of color to therapeutic cancer trials. The framework focused on refining the organizational culture and establishing an infrastructure that reinforced these changes, including a policy of disease‐specific accrual targets by race and gender. Clinicians were given benchmarks, and their accrual rates were tracked and documented for monitoring and feedback. Accrual of racial and ethnic minoritized groups was also monitored throughout trial implementation, and a process for communicating feedback to investigators about accrual was formalized. Between 2005 and 2010, there was a small but nonsignificant increase in the proportion of patients of color enrolled in clinical trials, from 12% to 14%, while patients of color represented about 17.5% of the total cancer patient population in treatment at the center during this time frame.

Finally, a clinical trial initiative was implemented at a prostate cancer clinic^42^ to inform eligible patients of clinical trial opportunities and provide more detailed treatment recommendations. A practice nurse helped facilitate a discussion about receiving treatment in a clinical trial at specialist visits and provided patients with a summary of their recommendations, including clinical trial options. Between 2004 and 2008, 1370 men with localized prostate cancer were seen at the cancer clinic, 24% of whom were seen before the initiative began. Enrollment in treatment trials increased from 6% to 15%; enrollment in procedural trials decreased from 8% to 2%.

### 4.4 Collaborative approaches

In an attempt to increase adolescent and young adult (ages 15–40 years) access to clinical trials, a children's hospital and adult cancer program in an urban area developed a collaborative program.^43^ Adult and pediatric clinicians provided consultation on patients, multidisciplinary meetings were held quarterly where pediatric and adult teams shared information about available trials, and the teams had a shared institutional review board. Clinical trial enrollment from 2006 to 2010 through the initiative was compared with historical enrollment data from the partner institutions from 2003 to 2006. Among adolescent and young adult patients in the program who were treated at the adult hospital, trial enrollment increased from 4% to 32% (*p* < 0.001). No increase was seen among those treated at the children's hospital.

## 5 INTERVENTIONS TARGETING PATIENT BARRIERS

We identified six studies that focused on interventions intended to reduce barriers at the patient level (Table 2), four of which were RCTs and two were retrospective pre‐post analyses. Only one of these studies specified the type of cancer (breast).^44^ Studies either focused on implementing educational tools at the patient level, generally using multiple media, or on the role of lay health workers in educating patients. Although the two pre‐post studies reported significant increases in accrual, the RCT data did not demonstrate improvements.

**TABLE 2:** Studies targeting patient barriers.

Study/Type/*N*	Patient population/Setting	Comparison/intervention	Results
Kamen et al.^45^ RCT *N* = 418	Patients with various types of cancer recruited nationwide by research coordinators at community‐based oncology clinics (including breast, lung, colon, and prostate) who were eligible to participate in a therapeutic cancer clinical trial	Psychoeducational intervention focused on changing attitudes about clinical trials, comparing a DVD plus print booklet with an informational print booklet alone	Clinical trial participation: DVD + booklet Yes: 68.8% No: 24.7% Undecided: 6.5% Booklet only Yes: 62% No: 36% Undecided: 2% *p* = 0.01 (chi square)
Skinner et al.^46^ RCT *N* = 63	Patients from urology, hematology, and breast clinics at a comprehensive cancer center; oversampled for racial/ethnic minorities	In‐office educational video about clinical trials versus video to take home and view	Clinical trial enrollment at 1 year: In‐office video: 3/37 No in‐office education: 2/42 *p* = 0.69
Felicitas‐Perkins et al.^47^ RCT *N* = 37	Filipino cancer patients in a community‐based oncology clinic who had a clinical trial available to them (no specific information provided about eligibility)	Language‐concordant DVD on clinical trial participation plus usual education versus usual education alone	23/37 eligible for a clinical trial Clinical trial enrollment among eligible patients: Usual education: 2/11 (18%) DVD + usual education: 3/12 (25%) *p* > 0.99
Robinson et al.^44^ Retrospective pre‐post *N* = 200	Black patients with breast cancer, Stages I–III or metastatic at hospitals that were part of a hospital system in the Northeast United States	15‐min culturally targeted video designed to affect attitudes of Black patients with cancer toward clinical trial participation	27/200 (13.5%) participants enrolled in a study within 6 months, a 7.5% increase in participants from 6% at baseline *p* < 0.001
Borno et al^59^ RCT *N* = 132	Patients at two NCI‐designated comprehensive cancer centers who were being approached to consider participation in a clinical trial	Language‐concordant (English, Spanish, and Chinese) phone call plus brochure to facilitate participation in a financial reimbursement program (FRP) versus brochure only (note: FRP was available to all)	Clinical trial enrollment was the same (70%) in both groups; the most common (75%) reason for not enrolling was ineligibility identified during screening
Patel et al.^48^ Retrospective pre‐post *N* = 138 (intervention + historical control)	Racial and ethnic minority, low‐income adult union workers in Chicago newly diagnosed with cancer	QI initiative with lay health workers focused on goals of care including education about the purpose and importance of clinical trials	72% of members eligible for trial who participated in 6‐month post‐intervention consented and enrolled versus 22% in the 6‐month pre‐intervention *p* < 0.001

Abbreviations: FRP, Financial Reimbursement Program; NCI, National Cancer Institute; QI, quality improvement.

### 5.1 Multimedia educational tools

Three RCTs and one pre‐post study used multimedia education to provide information to patients intended to increase participation in cancer clinical trials. None of the RCTs demonstrated increases in accrual; only the pre‐post study reported a significant increase of 7.5%.

Kamen et al.^45^ compared a multimedia psychoeducational intervention with a traditional print (control) intervention in 418 patients from National Cancer Institute (NCI) Community Oncology Research. Patients in the intervention group were given the intervention DVD to review, while the patients in the control group were given the standard print materials. At 2 months the proportion of patients in each arm who had opted to enroll in a clinical trial was 117/170 (69%) in the multimedia arm versus 119/192 (62%) in the print arm. A chi‐square analysis comparing those who enrolled with either those who declined or who were undecided suggested that there was a significant difference between the groups; however, when the comparison was between enrollees and a combined group of those who were undecided or who declined, there was no significant difference.

The second RCT compared patients at a comprehensive cancer center who viewed a clinical trial educational video in the office with patients who took the same video home.^46^ The video provided information on clinical trials, including benefits and risks, as well as personal stories from patients and oncologists. One year later, a very small number of participants overall were enrolled in a cancer clinical trial (3/37 in the video intervention group and 2/42 in the usual care group) (*p* = 0.69).

The third RCT piloted an education video to evaluate a language‐concordant multimedia educational tool among 37 Filipino cancer patients in Hawai‘i.^47^ The DVD intervention addressed knowledge about clinical trials, including safety, benefits, and the consent process. The video was presented in two languages (Tagalog and Ilokano) with English subtitles and found no difference in enrollment in the two groups. When patients did decline participation in a trial after the educational video, it was most often because there was a language barrier in the enrollment process itself.

In addition to the RCTs described above, Robinson^44^ describes a pre‐post evaluation of a 15‐minute video intervention with the primary outcome being signing informed consent documents or enrolment in a therapeutic trial within 6 months of intervention. The video addressed six key attitudes toward clinical trials identified among breast cancer patients who are Black. Two hundred female patients who are Black were shown the video. After the intervention, 39 (19.5%) patients consented to or enrolled in a therapeutic trial within 6 months, and 27 (13.5%) were enrolled in a study compared with 6% at baseline (*p* < 0.001).

### 5.2 Telephone outreach

Based on the potential for financial reimbursement to support clinical trial accrual, an RCT of telephone outreach compared with the usual pamphlet about available financial reimbursement was conducted in a population of patients who all had financial support available to them.^16^ Both groups received a pamphlet, but one group received an additional call about the program. There were no differences in clinical trial enrollment (70% in both arms).

### 5.3 Lay health workers

Finally, Patel et al.^48^ describe the impact of an intervention using lay health workers directly engaging with racial and ethnic minority union member patients from low‐income households who were diagnosed with cancer (nonspecified). Lay health workers assisted patients after their diagnosis and through the process of discussing the care and symptom burden with cancer teams, including educating them about clinical trial participation. The study compared a sample of 66 patients newly diagnosed with cancer with a historical cohort of 72 patients in the 6 months prior to the intervention. Most participants were Black or African American (45% in the intervention group; 46% in the control group) or Asian Pacific Islander (24% in the intervention group; 25% in the control group). More patients after implementation of the intervention enrolled in clinical trials (72%) compared with patients in the 6 months prior to the intervention (22%) (*p* < 0.001).

## 6 INTERVENTIONS TARGETING CLINICIANS AND RESEARCH STAFF

Only one study described an intervention specifically targeting clinician‐level barriers (Table 3), using a cultural competency training for clinical research staff and physician investigators. This pilot study of a 4‐h in‐person or virtual cultural competency training found no impact on accrual of racial and ethnic minority patients into clinical trials.^49^ Although the study was directed at both physicians and research staff, only 3% of the volunteers who participated were physicians; the rest were research staff. Accrual in clinical trials was compared at the clinic level between clinics where any individual had participated in training and those that had no volunteer participants. No differences were observed either pre‐post at individual centers or between centers.

**TABLE 3:** Interventions targeting clinicians and research staff.

Study/Type/*N*	Patient population/Setting	Comparison/intervention	Results
Wells et al.^49^ Quasi‐experimental pre‐post N of patient population unknown 67 clinical research associates (CRAs) and physicians were trained; 3% were physicians	Patients eligible to participate in a study of the NCI‐sponsored RTOG; types of cancer not specified	Cultural competency and recruitment training that focused on barriers, myths, beliefs, and norms within Latino and African American culture. Sites that included participants in the training were compared with sites where no clinical research associates or physicians participated	Racial and ethnic minority enrollment as a percentage of total site‐level enrollment did not change significantly after training and did not differ between sites with and without trainees

Abbreviations: NCI, National Cancer Institute; RTOG, Radiation Therapy Oncology Group.

## 7 POLICY INTERVENTIONS

We identified one policy analysis that met our criteria for inclusion (Table 4).

**TABLE 4:** Study of policy‐level change.

Study	Patient population	Comparison/intervention	Results
Ellis et al.^50^ Policy study	Community‐based practices in the NCI Community Clinical Oncology Program	State‐mandated insurance coverage for trial‐related costs	No effect

Abbreviation: NCI, National Cancer Institute.

Ellis et al.^50^ studied the effect of state mandates requiring insurance companies to cover clinical trial costs on patient enrollment in 37 states over 17 years from 1991 and 2007. The analysis compared clinical trial participation through 85 of NCI's Community Clinical Oncology Programs in states with and without mandates. Of 37 states, 13 contributed data both before and after implementation. There was no observed effect of policy mandates, including in multiple sensitivity analyses designed to account for other potential differences, although accrual did increase across all sites, suggesting that other factors were associated with these increases.

## 8 DISCUSSION

This scoping review aimed to identify evidence of strategies that increase accrual, defined as the number of cancer patients enrolled in therapeutic cancer clinical trials or the proportion of eligible patients enrolled in trials. We required that studies specify the time frame over which accrual was measured. This review stands in contrast to prior reviews that do not hold an increase in accrual as the standard for success, specifically accrual relative to baseline levels or compared with other interventions.

Based on the results of this review, it is difficult to identify with certainty any evidence‐based approaches that effectively increase accrual in cancer clinical trials, overall or for any particular population group. This is despite a large literature base identifying numerous barriers to participation at multiple levels, along with numerous recommendations and reports about how mitigating these barriers could lead to increased accrual.^16^, ^25^, ^51^, ^52^, ^53^, ^54^, ^55^ Many studies captured in our initial search were eliminated at the abstract phase because their outcome was not accrual into cancer treatment trials or did not provide data that could be used to verify whether an intervention had an effect. No studies were available to determine whether any interventions were more or less effective among patients clinical underrepresented in cancer treatment trials, including people from ethnic and racial minoritized groups, people with low incomes, those who live in rural areas, people who are aged 70 years and older, and adolescents and young adults.

The few studies that do report positive outcomes suffer from a lack of rigorous methods, most notably a lack of comparative methods appropriate for measuring effectiveness, as well as no attempt to enroll a representative sample or to include all eligible patients. Most also provided little detail about the resources necessary to implement the intervention. Many are not specific about the patient population and fail to provide even baseline numbers for the denominator. Strength of study design was inconsistent, and RCTs were only available in patient‐level interventions. Most studies included in this review were small, and results for a given intervention were rarely replicated, limiting the strength of evidence for any one intervention and making it impossible to draw firm conclusions about any one intervention being effective or ineffective.

Despite a lack of methodological rigor across most of the studies, those studies that reported positive outcomes generally were those at a system level and included actively prescreening patients and matching them to clinical trials. Having nurses provide tailored summaries of information on clinical trial availability and eligibility to both patients and providers was reported to be associated with increased trial accrual in a pre‐post study.^37^, ^42^, ^43^ Though presentation at a tumor board was associated with increased likelihood of enrollment in a therapeutic trial, the decision to present at a tumor board is inevitably at the clinician's discretion, so those cases are more likely to be considered for a trial from the outset and any reported benefit is confounded.

Patient trial matching, based on active prescreening at the system level has also been reported to increase accrual.^38^, ^42^ However, lack of methodological rigor, including a lack of comparative methods, makes it impossible to determine the degree to which such interventions were effective. Approaches that include matching patients to trials require investments in staff resources, both in terms of dedicated staff and time, as well as methods to communicate, and it also is important that reporting on future research in this space better describe the level of resources necessary to achieve success.

The use of lay health workers to assist patients after their diagnosis and through the process of discussing care and symptom burden with cancer teams also was reported to be associated with increases in clinical trial accrual.^48^ Although the study was small (*n* = 66), the benefits of providing a community health worker were realized not only in increasing accrual but also in documentation of goals of care and participation in palliative care. Other systematic reviews have documented the effectiveness of patient navigator interventions at improving clinical trial accrual, especially among patients from ethnic and racial minoritized groups,^56^, ^57^ but studies in these reviews were not published within our review time frame.

Although barriers to clinical trial enrollment among cancer patients are multilevel and systemic, a number of approaches to increasing accrual in clinical trials over the past 10 years have continued to focus on changing patient behavior, primarily through education of the individual patient.^58^ Interventions that focused on patient education made up a large proportion of studies in this review (31%); most studies compare modes of delivery of the same information^45^, ^46^ or tailor patient education to specific cultures and languages,^44^, ^47^, ^59^ and were predominantly ineffective.

As Unger and colleagues have suggested, the ongoing focus on interventions to address patient barriers suggests that patients themselves are the primary factor limiting trial enrollment and may well be a missed opportunity. Their systematic review and meta‐analysis found that system‐, institutional‐, and clinician‐level factors may have a much greater influence on patient participation.^4^ They conclude that the root causes of low participation in cancer treatment trials are due to structural and clinical barriers rather those associated with patient willingness or attitudes. Because many of these barriers are potentially modifiable, mitigating those barriers represents an “enormous opportunity to increase trial participation rates.”

Few studies focused exclusively on provider‐ and policy‐level interventions, although some multicomponent interventions included provider elements. The studies included in this review that did focus on provider‐ and policy‐level interventions found limited or no impact on accrual, had significant limitations, or were outdated. Though Wells et al.^49^ focused on improving cultural competency among clinicians, only 3% of those trained were physicians. The one policy study, by Ellis et al.,^50^ that our review identified found no effect in community oncology programs over several decades of state‐level mandates for insurers to provide support for routine costs of care on a clinical trials and is likely not applicable in the current policy environment (e.g., the Affordable Care Act and Clinical Treatment Act) as it was limited to 1991 to 2007. Though Borno et al.^59^ included a financial reimbursement component, the financial reimbursement program was available to all patients in the study, so the role of the program itself was not evaluable. Additionally, other factors that affect access to trials such as proximity to facilities conducting clinical trials, equitable offers of clinical trial participation, access to transportation, as well as social determinants of health were not addressed in the studies included in this review.

If we are to improve accrual in cancer treatment trials, we must have evidence‐based approaches to inform our recruitment and retention efforts. It is critical that research on interventions that aim to increase accrual employ rigor in study design. Future studies should use directly comparative designs, and RCTs where possible. Studies should ensure that interventions are fully described and that quantitative data are available to make clear inferences about the effect of the intervention in terms of numbers and proportions of participants accrued. Finally, studies should address more clearly any potential confounding.

### 8.1 Limitations

There were several limitations to our scoping review, both in terms of the design of the review and in the studies available. In limiting our review to studies published in the past 10 years that provided comparative data, either pre‐ and post‐ a clearly described intervention, or compared with another intervention, we did not include studies that compared cancer treatment trial accrual rates to national statistics, expected accrual rates or rates from other studies. Our requirement that studies must have included comparison data to demonstrate a difference in accrual rates was notably more stringent than other reviews, but necessary to establish the effectiveness of any interventions. Therefore, some potentially beneficial interventions may have been excluded. Our exclusion of studies published prior to 2012 may have excluded earlier studies that have demonstrated benefit, such as clinical trial navigator programs. Our decision to focus on studies conducted in the United States may have excluded successful approaches used in other countries; however, given the siloed nature of cancer care, the lack of universal health care make the US unique. Moreover, the United States is one of the most racially and ethnically diverse countries in the world; the intersection of different layers of disadvantage and how they impact clinical participation may also be unique to the United States. Finally, our review's reliance on the studies' use of the term “accrual” as defined by the NCI^60^ may have affected our conclusions, as it is not defined consistently across investigators, specifically, whether the investigators considered screen failures, participant enrollment, participant randomization, retention, or completion in their use of the term.

## 9 CONCLUSIONS

Clinical trials are vital for advancing cancer discoveries and are considered high quality cancer care, yet very few participate. Moreover, those that do take part are much less diverse than the population of people with cancer.^61^ If we are to improve the quality of cancer care, it is imperative that we increase overall participation in cancer treatment trials and develop and utilize evidence‐based approaches to inform our recruitment and retention efforts.

Despite the robust literature on the barriers to cancer treatment trial participation, often accompanied by promising recommendations or studies suggesting particular strategies, there is a lack of methodologically rigorous research that demonstrates which interventions increase accrual. This same conclusion was reached more than 10 years ago by the National Cancer Institute and the American Society of Clinical Oncology.^53^ We also note similar conclusions in other systematic reviews of clinical research^62^, ^63^, ^64^ in other diseases.

In addition, results of specific tested interventions were never replicated, limiting the strength of evidence for any one intervention and making it impossible to draw firm conclusions regarding efficacy. Finally, no studies assessed the degree to which interventions were effective in increasing accrual among underrepresented groups of patients as compared to other groups.

In conclusion, there is a paucity of high‐quality evidence to guide efforts to increase participation in cancer treatment clinical trials. More evidence must be generated to identify which interventions can be effective and for which populations of patients, and what resources are required to replicate them. Moreover, terminology must be standardized in this field of study (e.g., accrual, randomized, enrolled, participation, and completion). It is imperative that strategies for increasing participation in cancer clinical trials be developed and rigorously evaluated so that these strategies can be disseminated, participation in trials can increase and become more equitable, and trial results can become more generalizable. By utilizing evidence‐based strategies, we can enhance the speed with which clinical researchers can determine results and bring new treatments to patients who need them.

## AUTHOR CONTRIBUTIONS

**Margo Michaels:** Conceptualization (lead); investigation (equal); writing – original draft (equal); writing – review and editing (equal). **Elisa S. Weiss:** Conceptualization (lead); investigation (equal); writing – original draft (equal); writing – review and editing (equal). **Maria Sae‐Hau:** Conceptualization (lead); investigation (equal); writing – original draft (equal); writing – review and editing (equal). **Dora Illei:** Data curation (equal); formal analysis (equal); project administration (equal); visualization (equal); writing – original draft (equal). **Bethany Lilly:** Conceptualization (equal); writing – review and editing (supporting). **Leah Szumita:** Conceptualization (equal); writing – review and editing (supporting). **Brian Connell:** Conceptualization (supporting); writing – review and editing (supporting). **Marialanna Lee:** Conceptualization (supporting); writing – review and editing (supporting). **Eric Cooks:** Conceptualization (supporting); writing – review and editing (supporting). **Melissa McPheeters:** Conceptualization (equal); data curation (lead); formal analysis (equal); funding acquisition (lead); investigation (lead); methodology (lead); project administration (lead); supervision (lead); writing – original draft (lead); writing – review and editing (equal).

## FUNDING INFORMATION

This work was funded in part under a contract from the Leukemia and Lymphoma Society to RTI International that supported effort for the RTI International team members.

## CONFLICT OF INTEREST STATEMENT

No authors report any conflicts of interest.

## PRECIS

Despite extensive literature about the barriers that impede cancer treatment trial accrual, results reveal little evidence published in the last 10 years on interventions that increase accrual.

## Data Availability

No new data were generated or analyzed for this scoping review.

## SEARCH STRATEGY AND YIELD

### 1

Search Conducted August 5, 2022.

Topic: A scoping review on what approaches have been effective in increasing accrual into clinical trials for cancer. There are systematic reviews about barriers, but few on the effectiveness of interventions/approaches for addressing those barriers.

Language: English.

Years: 2012‐present.

Population: Adults in high index countries.

Database: PubMed/MEDLINE, Embase, CINAHL, Web of Science.

Keywords:

(“clinical trial accrual” OR “clinical trial enrollment” OR “clinical trial enrollment barrier*” OR “enrollment in clinical trials” OR “patient participation in clinical trials”) AND cancer.

Total Unique (duplicates removed with EndNote) Records – 2098.

**Table:**

PubMed—1052 records
Cancer Terms
#1 (cancer*[Title] OR oncolog*[Title] OR “Neoplasms”[Mesh] OR “Antineoplastic Agents”[Mesh]) AND (“2012/01/01”[Date – Publication]: “3000”[Date – Publication]) Filters: English 1,488,294
Trial Terms
#2 (trial*[Title] OR “Clinical Trials as Topic”[Mesh]) AND (“2012/01/01”[Date – Publication]: “3000”[Date – Publication]) Filters: English 294,130
Accrual Terms
#3 (“accrual”[Title] OR enroll*[Title] OR participat*[Title] OR “clinical trial accrual”[Title/Abstract] OR “clinical trial enrollment”[Title/Abstract] OR “enrollment barrier*”[Title/Abstract] OR “enrollment in clinical trials”[Title/Abstract] OR “participation in clinical trials”[Title/Abstract] OR (“Patient Participation”[Mesh] AND “Clinical Trials as Topic”[Mesh] AND “Neoplasms”[Mesh])) AND (“2012/01/01”[Date – Publication]: “3000”[Date – Publication]) Filters: English 26,480
Total
#4 (#1 AND #2 AND #3) AND (“2012/01/01”[Date – Publication]: “3000”[Date – Publication]) Filters: English 1137
Exclusions
#5 (#4 NOT ((“Animals”[Mesh] NOT “Humans”[Mesh]) OR ((“Child”[Mesh] OR “Infant”[Mesh]) NOT (“Adult”[Mesh] OR “Adolescent”[Mesh])) OR “Comment”[Publication Type] OR “Letter”[Publication Type] OR “Editorial”[Publication Type])) AND (“2012/01/01”[Date – Publication]: “3000”[Date – Publication]) Filters: English 1052

Embase—1505 records
Cancer Terms
#1 cancer*:ti OR oncolog*:ti OR ‘malignant neoplasm’/exp/mj OR ‘antineoplastic agent’/exp/mj AND [english]/lim AND [2012–2022]/py 1,897,795
Trial Terms
#2 trial*:ti OR ‘clinical trial (topic)’/exp/mj AND [english]/lim AND [2012–2022]/py 271,140
Accrual Terms
#3 ‘accrual’:ti OR enroll*:ti OR participat*:ti OR ‘clinical trial accrual’:ti,ab OR ‘clinical trial enrollment’:ti,ab OR ‘enrollment barrier*’:ti,ab OR ‘enrollment in clinical trials’:ti,ab OR ‘participation in clinical trials’:ti,ab OR (‘patient participation’/exp/mj AND ‘clinical trial (topic)’/exp/mj AND ‘malignant neoplasm’/exp/mj) AND [english]/lim AND [2012–2022]/py 25,506
Total
#4 #1 AND #2 AND #3 AND [english]/lim AND [2012–2022]/py 1588
Exclusions
#5 #4 NOT ((‘animal’/exp NOT ‘human’/exp) OR ((‘child’/exp OR ‘infant’/exp) NOT (‘adult’/exp OR ‘adolescent’/exp)) OR comment*:ti OR ‘letter’/exp OR ‘editorial’/exp OR letter:it OR editorial:it) AND [english]/lim AND [2012–2022]/py 1505

CINAHL—246 records
Cancer Terms
S1 TI cancer* OR TI oncolog* OR MH “Neoplasms” OR MH “Antineoplastic Agents+” Limiters – Published Date: 20120101–20221231; English Language; Exclude MEDLINE records 143,544
Trial Terms
S2 TI trial* OR MH “Clinical Trials+” Limiters – Published Date: 20120101–20,221,231; English Language; Exclude MEDLINE records 89,106
Accrual Terms
S3 TI “accrual” OR TI enroll* OR TI participat* OR TI “clinical trial accrual” OR TI “clinical trial enrollment” OR TI “enrollment barrier*” OR TI “enrollment in clinical trials” OR TI “participation in clinical trials” OR AB “clinical trial accrual” OR AB “clinical trial enrollment” OR AB “enrollment barrier*” OR AB “enrollment in clinical trials” OR AB “participation in clinical trials” OR (MH “Consumer Participation” AND MH “Clinical Trials+” AND MH “Neoplasms”) Limiters – Published Date: 20120101–20221231; English Language; Exclude MEDLINE records 11,602
Total
S4 S1 AND S2 AND S3 Limiters ‐ Published Date: 20120101–20,221,231; English Language; Exclude MEDLINE records 272
Exclusions
S5 S4 NOT ((MH “Animals+” NOT MH “Human”) OR ((ZG “child, preschool: 2–5 years” OR ZG “child: 6–12 years” OR ZG “infant, newborn: birth‐1 month” OR ZG “infant: 1–23 months” OR MH “Child+” OR MH “Infant+”)) NOT ((ZG “adolescent: 13–18 years” OR ZG “adult: 19–44 years” OR ZG “aged, 80 & over” OR ZG “aged: 65+ years” OR ZG “middle aged: 45–64 years” OR MH “Adult+” OR MH “Adolescence+”)) OR ZT “commentary” OR ZT “editorial” OR ZT “letter” OR ZT “letter to the editor”) Limiters ‐ Published Date: 20120101–20221231; English Language; Exclude MEDLINE records 246

Web of Science—1166 records
Cancer Terms
#1 TI = (cancer* OR oncolog*) OR AK = (cancer* OR oncolog*) and English (Languages) Timespan: 2012‐01‐01 to 2022‐12‐31 (Publication Date) 1,091,411
Trial Terms
#2 TI = (trial*) OR AK = (trial*) and English (Languages) Timespan: 2012‐01‐01 to 2022‐12‐31 (Publication Date) 328,823
Accrual Terms
#3 TI = (“accrual” OR enroll* OR participat* OR “clinical trial accrual” OR “clinical trial enrollment” OR “enrollment barrier*” OR “enrollment in clinical trials” OR “participation in clinical trials”) OR AB = (“clinical trial accrual” OR “clinical trial enrollment” OR “enrollment barrier*” OR “enrollment in clinical trials” OR “participation in clinical trials”) and English (Languages) Timespan: 2012‐01‐01 to 2022‐12‐31 (Publication Date) 64,111
Total
#4 #1 AND #2 AND #3 and English (Languages) Timespan: 2012‐01‐01 to 2022‐12‐31 (Publication Date) 1291
Exclusions
#5 #4 NOT (TI = (“Animal*” NOT “Human*”) OR TI = ((“Child*” OR “Infant*”) NOT (“Adult*” OR “Adolescent*” OR teen* OR youth*))) and English (Languages) and News Item or Correction or Letter or Editorial Material (Exclude – Document Types) Timespan: 2012‐01‐01 to 2022‐12‐31 (Publication Date) 1166
